# Comparison of haematological parameters determined by the Sysmex KX - 2IN automated haematology analyzer and the manual counts

**DOI:** 10.1186/1472-6890-10-3

**Published:** 2010-04-23

**Authors:** Samuel O Ike, Thomas Nubila, Ernest O Ukaejiofo, Imelda N Nubila, Elvis N Shu, Ifeyinwa Ezema

**Affiliations:** 1 Department of Medical Laboratory Sciences, Faculty of Health Siences And Technology, College of Medicine, University of Nigeria, Enugu Campus, Enugu State, Nigeria; 2 Department of Medicine, College of Medicine, University of Nigeria, Enugu Campus, Enugu State, Nigeria; 3 Department of Pharmacology and Therapeutics, College of Medicine, University of Nigeria, Enugu Campus, Enugu State, Nigeria

## Abstract

**Background:**

This study was designed to determine the correlation between heamatological parameters by Sysmex KX-21N automated hematology analyzer with the manual methods.

**Method:**

Sixty (60) subjects were randomly selected from both apparently healthy subjects and those who have different blood disorders from the University of Teaching Hospital (UNTH), Ituku-Ozalla, Enugu, Enugu State, Nigeria. Three (3)mls of venous blood sample was collected aseptically from each subject into tri-potassium ethylenediamine tetra-acetic acid (K_3_EDTA) for the analysis of haematological parameters using the automated and the manual methods.

**Results:**

The blood film report by the manual method showed that 50% of the subjects were normocytic-normochromic while the other 50% revealed different abnormal blood pictures. Also, there were statistically significant differences (p < 0.05) in mean cell hemoglobin concentrations (MCHC) between the two methods. Similarly, the mean (S.E) values of hemoglobin, packed cell volume, platelet and total white cell counts demonstrated statistically significant difference (p < 0.001) and correlated positively when both methods were compared.

**Conclusion:**

From the present study, it can be concluded that the automated hematology analyzer readings correlated well with readings by the standard manual method, although the latter method gave additional diagnostic information on the blood pictures. While patients' care and laboratory operations could be optimized by using manual microscopic examination as a reflective substitute for automated methods, usage of automated method would ease our workload and save time for patients.

## Background

The automated hematology analyzer with complete blood count (CBC) results has replaced the traditional manual or individual assay methods for haematological parameters and the eye count leucocyte differential as the initial screening and detection system for haematological abnormalities in modern hospitals and clinics[1]. The traditional review of all automated hematology instrument results by preparation, staining and microscopic examination of a blood film examination has disappeared in most institutions[2]. The reasons are the more accurate detection of specimens with distributional or morphological abnormalities by the traditional eye count method[3].

The opportunity for a clinician to request a microscopic examination of a blood film, whether or not it is flagged, must be preserved, because the clinician's knowledge of the patient's history, physical findings, and current or prior therapy may indicate review to discover an abnormality that may not have been apparent from the instrument results alone. There has also been a dramatic reduction of the numbers of medical technologists and technicians in medical laboratories. Automated complete blood count and differential counts has reduced the number of technologists needed for performance of these tests[4]. But other factors have had a negative effect, such as the necessity to reduce costs. Consolidation of hematology and chemistry laboratories in core laboratories may produce savings in labor costs, but may also create problems of creating and maintaining areas of expertise, such as haematological morphology, because of cross-training required and the necessity of personnel to do all things[2].

Furthermore, hematology analyzers provide quick and accurate results in most situations. However, false results related either to platelets or other parameters from complete blood count may be observed in several instances, false low white blood cell (WBC) counts may be observed because of agglutination in the presence of ethylenediamine tetra-acetic acid (EDTA)[5].

Despite the sophistication of present day instruments, there is still need to depend on manual techniques for primary calibration. This highlights the importance of the need to maintain the manual technical skills, and to ensure this by appropriate technician training programme, despite the temptation to leave it all to the machines. Also, the correlation between automated hematology analyzer and manual techniques is rare and conflicting. Hence, this present study was designed and conducted to determine the relationship between Sysmex KX-21N automated hematology analyzer blood counts and manual counts using randomly selected human subject's blood samples at the department of hematology. UNTH, Ituku-Ozalla, Enugu, Enugu State, Nigeria.

## Methods

Venous blood samples were randomly collected from sixty (60) subjects. This comprised of both apparently healthy subjects (who came for medical examination) and those who have different blood disorders from University of Nigeria Teaching Hospital (UNTH), Ituku-Ozalla, Enugu, Enugu State, Nigeria. Ethical approval was obtained from the ethical Review Board of the University of Nigeria Teaching Hospital, Enugu. Consent was obtained from each subject at the commencement of the study.

The study was conducted in a routine hematology laboratory at the same hospital. Three (3) mls of blood sample was collected aseptically from each subject into tri-potassium ethylenediamine tetra-acetic acid (K_3_EDTA) anticoagulant bottle. This was well mixed by gentle inversion for complete blood count (CBC) analysis. Blood sample was divided into 2 parts as follows: Two (2) mls for manual method and one (1) ml for automated method using hematology auto analyzer Sysmex KX-21N.

One hundred (100) White blood cell (WBC) was counted by one competent and experienced medical laboratory scientist for both total and differential leucocyte counts. All manual samples were analyzed using standard hematological method as described by Dacie and Lewis[6], while the automated analysis was done following the manufacturer's operational guidelines. All samples were analyzed within 30 minutes of collection.

Haemoglobin (Hb) was estimated by the cyan-methaemoglobin method: Packed Cell Volume was estimated by the microhaematocrit method: Total and differential leucocyte counts were done by visual method. Mean Cell Haemoglobin Concentration (MCHC) was calculated from a knowledge of the haemoglobin and PCV.

### Statistical Analysis

The SPSS software was used in the statistical analysis. A p-value of less than 0.05 (p < 0.05) was considered significant.

## Results

The differential leucocyte count by the automated method showed four (4) blood samples with incomplete differential white cell count (<100 cells). Also, thirty (30) blood films demonstrated normochromic-normocytic (50%), 12 (20%) normocytic-hypochromic, 1(6.66%) microcytic-normochromic, (3.33%) increased platelet distribution, 2 (3.33%) decreased platelet distribution, 1 (1.66%) target cells, 1 (1.66%) polychromacia, 2 (3.33%) rouleaux, band form neutrophil and 10 (16.60%) demonstrated microcytic and hypochromic blood pictures. There were no reactive lymphocytes (Table 1).

**Figure 1:**
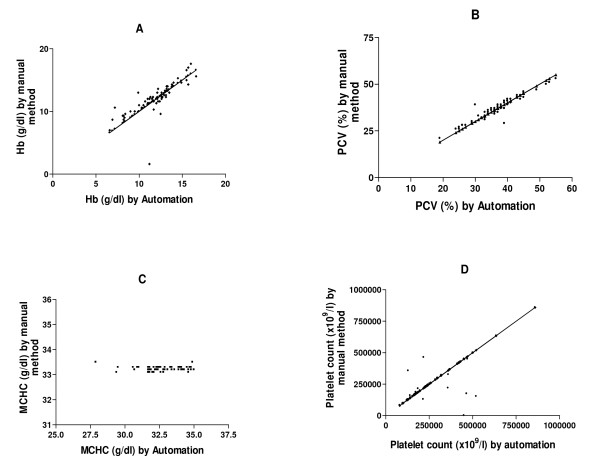
**The correlation of Hb, PCV, MCHC and Platelet count (A, B, C and D respectively) between automated method and manual method**.

**Figure 2:**
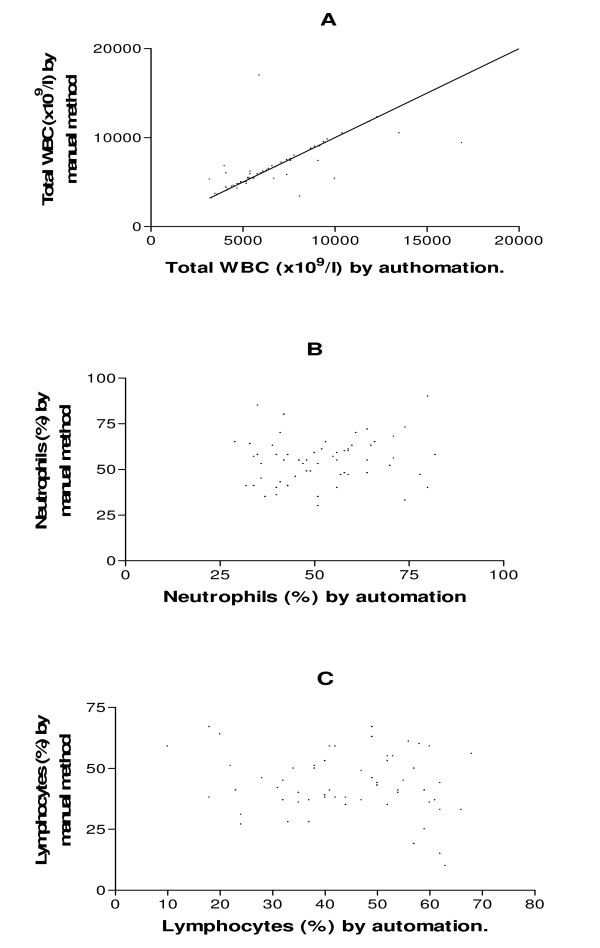
**The correlation of TWBC, Neutrophi l and Lymphocyte counts (A, B and C respectively) between automated method and manual method**.

Furthermore, hemoglobin, packed cell volume, platelet and total white cell counts only revealed statistically significant differences (p < 0.0001), and correlated positively (r = 0.7816, 0.9496, 0.7791 and 0.6717 respectively) when the mean and S.E values of the two methods (automated and manual) were compared (Figures 1 and 2).

**Table 1:** Manual microscopic peripheral blood film assessment in all the subjects studied.

PARAMETER	NUMBER (n)	PERCENTAGE (%)
Normocytic-normochromic	30	50
Normocytic-hypochromic	12	20
Macrocytic-normochromic	1	1.66
Microcytic-hypochromic	10	16.60
Polychromacia	1	1.66
Increased platelet	2	3.33
Decreased platelet	2	3.33
Target cells	1	1.66
Rouleaux formation and band form neutrophils	1	1.66

## Discussion

Automated peripheral blood, leucocyte counts (LDCs) are widely accepted in routine practice. However, many laboratories still reflexively perform manual LDCs based solely on abnormal automated results or instruments "flags", before any manual triage step[1]. In the present study, the differential leucocyte count by automated method demonstrated (4) "unflagged" samples with incomplete white cell count (<100 cells). This might probably be due to inability of the automated machine to identify/differentiate the leucocytes, more especially the immature cells as previously reported by Lewis *et. Al*,1999)[7]. Also, this is in agreement with an earlier report by Takubo and Tatsuni[8] whose result indicated discrepancies in a quality control (QC) survey in a manual leucocyte differential count which was attributed to poor differentiation of segmented neutrophils and band neutrophils.

The direct microscopic visualization of prepared blood film reported among the subjects (60) studied showed that 30 subjects (50%) were normocytic-normochromic, while the others (50%) revealed different abnormal blood pictures which are very important in the diagnosis of different blood disorders (Table 1). However, the imprecision in measurement of haemoglobin(Hb) and packed cell volume (PCV) by the manual method may result in variations in the Red blood cell (RBC) indices. This is best seen with mean cell haemoglobin concentration (MCHC) which may result in misclassification of values for diagnosis of the anaemias. This indicated that, although slow and at times cumbersome (Rock *et al*; 1984), the manual method still has some advantages over the automated methods.

The result of the present study is in contrast with an earlier report by Pierre[2] and Novis *et. al;*[3] who reported that automated haematology instruments are more accurate in the detection of specimens with distributional or morphologic abnormalities than by the traditional eye count method. However, the 100 cell count adopted in this present study could have contributed to some statistical variations observed between the automated and manual methods.

In addition, the mean (S.E) values of hemoglobin, packed cell volume, platelet and total white blood cell counts, revealed statistically significant differences (p < 0.0001) (Table 2) and correlated positively when both methods (automated and manual) were compared (Figures 1 and 2). This indicates that the automated hematology analyzer (Sysmex KX-21N) readings correlated well with the manual methods. This is in line with an earlier report by Atilola[9] and McCarthy *et. al *[10].

**Table 2:** Comparison of the mean ± S.E of hematological profile of automated method with the manual method.

PARAMETER	AUTOMATED (MEAN ± S.E)	MANUAL (MEAN ± S.E)
HB (g/dl)	11.86 ± 0.30	12.07 ± 0.33***
PCV (%)	36.00 ± 0.91	37.00 ± 0.83***
MCHC (g/dl)	32.80 ± 0.19	33.24 ± 0.01
PLATELET(×10^9^/l)	265.5 ± 18.94	251.7 ± 18.58***
TWBC(×10^9^/l)	7.00 ± 0.50	11.41 ± 4.90***
NEUTROPHILS (%)	52.15 ± 1.77	54.68 ± 1.61
LYMPHOCYTES	44.42 ± 1.79	43.48 ± 1.59

***Significantly different to value for automated method (p < 0.0001).

## Conclusion

The results of the present study confirm that the automated hematology analyzer readings are as reliable as the standard manual method even though the latter method gives additional diagnostic information through the blood pictures. Hence, manual microscopic blood examination should always be used to validate the automated methods as previously suggested by Lantis *et. al*[1]. Patients' care and laboratory operations should be optimized by using standard manual microscopic examination in conjunction with the automated methods, especially with respect to the differential leucocyte counts and blood cell morphology.

## Competing interests

The authors declare that they have no competing interests.

## Authors' contributions

**NT **conceived of the study and participated in its design and coordination. **EOU **participated in the design of the study and revising it critically for intellectual content.

**SOI **participated in the design of the study, revising it critically for intellectual content and drafting of the manuscript. **ENS **participated in the design of the study and performed the statistical analysis. **IJE **participated in the design of the study and carried out the sample collection and processing. All authors read and approved the final manuscript.

## Pre-publication history

The pre-publication history for this paper can be accessed here:

http://www.biomedcentral.com/1472-6890/10/3/prepub
